# Factor V Leiden, MTHFR, and FXIIIVal34Leu gene polymorphisms and their association with clinical features and risk of diabetic retinopathy in patients with type 2 diabetes

**DOI:** 10.22088/cjim.15.1.11

**Published:** 2024

**Authors:** Atefeh Rahimi, Nastaran Moridi, Amin Golestani, Gholamreza Anani-Sarab, Fatemeh Salmani, Gholamhossein Yaqubi, Behzad Mesbahzadeh, Mohammad Ali Jalalifar, Mohammad Malekaneh, Seyed Mehdi Sajjadi

**Affiliations:** 1 Student research committee, Birjand University of Medical Sciences, Birjand, Iran; 2 Cellular and molecular research center, Birjand University of Medical Sciences, Birjand, Iran; 3 Social Determinants of Health Research Center, Birjand University of Medical Sciences, Birjand, Iran; 4 Department of Ophthalmology, School of Medicine, Birjand University of Medical Sciences, Birjand, Iran; 5 Cardiovascular Diseases Research Center, Birjand University of Medical sciences, Birjand, Iran; 6 Thalassemia and Hemoglobinopathy Research Center, Ahvaz Jundishapur University of Medical Sciences, Ahvaz, Iran; 7 Department of Clinical Biochemistry, School of Medicine Birjand University of Medical Sciences, Birjand, Iran; # This author Contributed equally to this work as the first author

**Keywords:** Diabetic retinopathy, Factor V Leiden, MTHFR, Factor XIII, T-ARMS-PCR

## Abstract

**Background:**

Diabetic retinopathy (DR) is expanding to epidemic levels globally due to the progressing prevalence of diabetes mellitus (DM). In this study, the association between factor V Leiden (*FVL*), *MTHFR*C677T, and *FXIIIVal34Leu* polymorphisms and diabetic retinopathy was investigated in Eastern Iran.

**Methods:**

This case-control study enlisted the participation of 300 people (diabetic patients=100, diabetic retinopathy patients=100, healthy controls=100), and polymorphisms were examined by Tetra primer ARMS-PCR.

**Results:**

The frequency of *FVL* (p=0.294) and *FXIIIVal34Leu* (P=0.349) polymorphism showed no significant results between the genotype frequency in the mentioned groups. In contrast, *MTHFRC677T* SNP was significantly different in diabetic patients and controls (P=0.008). The MTHFRC677T polymorphism was found to be connected with increased systolic blood pressure in patients who had the TT genotype (130.96±11.92mm/Hg; P=0.011).

**Conclusion:**

Our study recommended that the *MTHFRC677T* polymorphism may offer to DR development. Studies with larger sample sizes and a wider spectrum of populations are authorized to verify this finding.

Diabetes, the most common metabolic disease, is the fifth leading cause of death globally. It has been reported that in 2017, 451 million people suffered from diabetes globally and this number is expected to rise up to 693 million by 2045 (1). The most common complications associated with diabetes include dyslipidemia, nephropathy, neuropathy, diabetic foot, stroke, and vascular problems. Diabetic retinopathy (DR), a microvascular disease, generally affecting 50% of patients (2). It is additionally considered the most common cause of vision loss in 20- to 60-year-olds. Since genetic factors are considered important in the occurrence of this disease, patient screening for the genetic mutations is suggested. The most common genetic disorder that leads to thrombosis is Factor V Leiden polymorphism (FVL), which increases the resistance of FV to activated protein C (APCR) (3). Methylenetetrahydrofolate reductase (MTHFR) is an enzyme that aids in the conversion of homocysteine to methionine by serving as a methyl group donor (4-7). 

Deficiency of this enzyme causes accumulation of homocysteine in the body, which leads to a functional defect in the endothelium and the development of a prothrombotic state. Numerous studies have shown C677T mutation to be related with type 2 diabetes as a vascular risk factor (4-7). Another factor that may play a role in vascular thrombosis is the factor XIII Val34Leu polymorphism, which is essential for stabilization of the fibrin network after the activation of fibrinogen by thrombin (8). 

Tetra primer ARMS-PCR (T-ARMS-PCR) approach uses four primers, two outer primers that are not allele-specific and two inner primers that are, but in the opposite direction from one another. The inner primers interact with the corresponding opposite outside primer to produce smaller allele-specific fragments, while the outer primers amplify a substantial portion of the target gene as an internal control during the reaction. It is important to note that the nested primers have a purposeful mismatch added to the 3' terminal end to improve allele specificity.

Due to the importance of these polymorphisms in the incidence of venous thrombosis, this study was conducted to investigate their association with diabetic retinopathy in Iranian diabetic patients using T-ARMS-PCR.

## Methods

**Study Population:** A total of 300 subjects, including 100 diabetic patients (type II) without retinopathy (55 men, 45 women; age range, 52.73±5.85 years old), 100 diabetic patients (type II) with retinopathy (50 men, 50 women; age range, 55.40±5.69 years old), and 100 healthy individuals (50 men, 50 women; age range, 52.23±5.66 years old) were enrolled from 2019–2020 in South-Khorasan province, Birjand, Iran. All individuals in the study were of the same ethnic background but from different families to evade the problem of allelic association. All subjects provided written informed permission in accordance with the principles outlined in the 1964 Helsinki declaration. The institutional ethics committee gave its approval to the study’s comprehensive procedure (IR.BUMS.REC.1398.310).

Inclusion criteria: Slit-lamp examination, funduscopy, and visual acuity that are all components of an ophthalmological examination, were performed by an ophthalmologist to confirm the disease. Blood samples of type 2 diabetes mellitus (T2DM) were collected after the diagnosis according to the American Diabetes Association Criteria with fasting blood glucose ≥126mg/dl, A1C >6.5%, and glucose tolerance test (GTT) >200mg/dl. Healthy individuals from the same geographic area who had no DR or family history of diabetes were considered for the study. 

Exclusion criteria: The study excluded patients with any concurrent coagulation disease, such as DVT, heart failure, previous angiography, and coagulation disease history. Patients on hemodialysis were also excluded from the study.

Measurements: All patients' demographic and clinical data, including as age, sex, body mass index (BMI), systolic and diastolic blood pressure, fasting blood sugar (FBS), and glycosylated hemoglobin (Hb AIc), two-hour post prandial (2hpp), levels of total cholesterol, high-density lipoprotein (HDL), low-density lipoprotein (LDL), triglyceride (TG), hemoglobin, liver function test (LFT), and creatinine were gathered. DNA Extraction: Following the manufacturer's recommendations, genomic DNA was isolated from whole blood using a Sina clone kit (Sina gene, Iran), and then kept at -20°C. An EpochTM Nano drop device was used to evaluate DNA quality and concentration (BioTek, USA).

SNP selection and genotyping: The primers were designed by Primer 1 (a web primer design program) (10) and prepared by the Metabion (Germany) (table 1). The genotypes of the patients were evaluated by the T-ARMS-PCR. The PCR reaction volume was 20μl, containing 10μl of 1X PCR master mix (Ampliqon Inc, Denmark), 1μl of genomic DNA (50-100ng/μl), 0.25μl of each outer primer, 1μl of each inner primer, and 6.5 μl of distilled water. PCR protocols are shown in table 2. Finally, using electrophoresis on a 2% agarose gel, the PCR results were examined, and the DNA bands were seen under a UV light source.

Statistical Analysis: IBM SPSS Version 16.0 was used to analyze the data. To compare groups, the mean and standard deviation (SD) of normally distributed data were computed, and the ANOVA test was applied. Genotype distribution and allele frequency between groups was compared by the χ2-test. Fisher's exact test was applied to determine the allele frequencies and genotypes in the various patient subdivisions. Logistic analysis was used to analyze the association between polymorphisms and the risk of diabetic retinopathy. Medians were used to express non-normally distributed data, and patients were compared using the Mann–Whitney U test. The threshold for statistical significance was p< 0.05.

**Table 1:** Primer sequences used for amplification of FVL, MTHFR, and FXIII gene polymorphisms

**SNP**	**Primer Sequence**	**Conc(μM)**	**Ta (◦C)**	**Product Size (bp)**
**FVL** **G1691A**	Forward inner primer (A allele): GAGCAGATCCCTGGACAGTCA	0.2 μM	57.1	Common: 242
	Reverse inner primer (G allele): ACTTCAAGGACAAAATACCTGTATTCATC			
	Forward outer primer (5' - 3'): GAACATCTTAGAGTTTGATGAACCCAC			G Allele: 175
	Reverse outer primer (5' - 3'): CCCATTATTTAGCCAGGAGACCTAA			A Allele: 117
MTHFR C677T*	Forward inner primer (T allele): TTGAAGGAGAAGGTGTCTGCGGGCGT	0.2 μM	65.0	Common: 407
	Reverse inner primer (C allele): CAAAGAAAAGCTGCGTGATGATGAAATAGG			
	Forward outer primer (5' - 3'): CCCAGCCACTCACTGTTTTAGTTCAGGC			C Allele: 273
	Reverse outer primer (5' - 3'): GGTGAGAGTGGGGTGGAGGGAGCTTAT			T Allele: 190
**FXIIIVal34Leu**	Forward inner primer (T allele): CTGCCCACAGTGGAGCTTCAGGACT	0.2 μM	69.1	Common: 414
	Reverse inner primer (G allele): TGACGCCCCGGGGCACTAC			
	Forward outer primer (5' - 3'): CGGCAAAATGTGTTGCTCAAGTGCT			G Allele: 268
	Reverse outer primer (5' - 3'): TAAAACCAGAGATTGGCAGGGGGCT			T Allele: 190

## Results

**Figure1:**
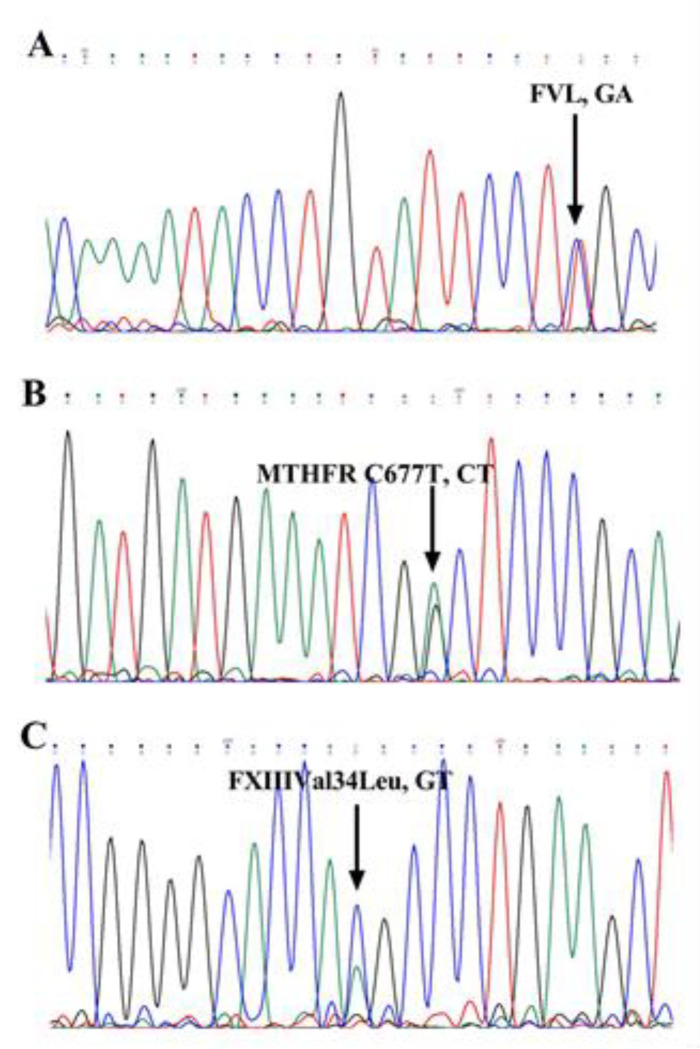
The results of DNA sequencing. Only heterozygous genotypes are shown. FVL (A), MTHFR C677T (B), and FXIIIval34leu (C)

The genotype of the *FVL*, *MTHFRC677T*, and *FXIIIVal34Leu *SNPs were determined in all individuals (table 3), which were then confirmed by sequencing in some samples (Fig.1). No significant differences were found between the genotype frequency of *FVL* (P = 0.294) and *FXIIIVal34Leu* (P = 0.349) among groups. Nonetheless, there was a substantial difference between the patients and controls for MTHFRC677T (P=0.008). Significant differences were found between the *TT* and *CC* genotypes of the diabetic and control groups, respectively (*TT* 16% vs. 5%; CC 43% vs. 62%). When the diabetic and diabetic retinopathy groups were compared, there was a remarkable difference only in the TT genotype (TT 16% vs. 6%). With exception to systolic blood pressure (table 5) (P=0.011), there were no meaningful association between *MTHFR C677T* polymorphism and other biochemical and hematological parameters that are presented in table 4. 

**Table 2:** . PCR amplification protocols for FVL, MTHFR, and FXIII gene polymorphisms detection

	**FVL G1691A**	**MTHFR C677T**	**FXIIIVal34Leu**	
**Initial Denaturation**	95^◦^C- 5min	94^◦^C- 5min		95^◦^C- 5min
**Denaturation**	95^◦^C-30s	94^◦^C-1min		95^◦^C-30s
**Annealing**	57.1^◦^C- 25s 25cycle	65^◦^C- 45s 30cycle		69.1^◦^C- 25s 33cycle
**Extension**	72^◦^C- 30s	72^◦^C- 45s		72^◦^C- 30s
**Final Extension**	72^◦^C- 10min	72^◦^C- 5min		72^◦^C- 10min

**Table 3:** The frequency distributions of the FVL, FXIIIVal34Leu, and MTHFR polymorphism alleles among patients and controls

**Polymorphisms**	**Genotype**	**Diabetic**		**Diabetic Retinopathy**		**Control**		**P-value**	**OR**	**95% CI**
		Number	Percent	Number	Percent	Number	Percent			
**FVL**	GG GA AA	97 3 0	97 3 0	97 2 1	97 2 1	100 0 0	100 0 0	0.375 0.377 1.000	0.000	0.000
									4.996	0.000
									1.000	0.000
**Allele**	G A	197 3	98.5 1.5	196 4	98 2	200 0	100 0	0.375 1.000		
**FXIIIV34L**	GG GT TT	73 25 2	73 25 2	78 20 2	78 20 2	66 29 5	66 29 5	0.163 0.361 0.520	1.393	0.761-2.551
									0.816	0.437-1.526
									0.388	0.073-2.047
**Allele**	G T	171 29	85.5 14.5	176 24	88 12	161 39	80.5 19.5	0.170 0.520		
**MTHFRC677T**	CC CT TT	43 41 16	43 41 16	49 45 6	49 45 6	62 33 5	62 33 5	0.024 0.212 0.013	0.462	0.263-0.814
									1.411	0.793-2.512
									3.619	1.271-10.303
**Allele**	C T	127 73	63.5 36.5	143 57	71.5 28.5	157 43	78.5 21.5	0.024 0.013		

OR: odds ratio, CI: Confidence Interval

**Table 4:** The demographic and clinical features of the subjects

**Risk Factor**	**Diabetic**	**Diabetic Retinopathy**	**Control**	**P-value**
	**Mean**	**Mean**	**Mean**	
**Age (year)**	**52.73**	**55.40**	**52.21**	**>0.05**
**BMI (kg/m2)**	**26.52**	**26.91**	**28.42**	**<0.05**
**FBS (mg/dl)**	**166.28**	**161.18**	**90.45**	**0.445**
**A1C (%)**	**8.02**	**8.36**	**5.06**	**0.285**
**2hpp (mg/dl)**	**242.49**	**242.25**	**118.67**	**0.989**
**SP (mmHg)**	**129.9**	**126.46**	**120.04**	**0.392**
**DP (mmHg)**	**74.61**	**79.54**	**80.01**	**0.153**
**Chol (mg/dl)**	**160.15**	**188.59**	**152.04**	**0.063**
**TG (mg/dl)**	**145.59**	**163.06**	**149.52**	**0.087**
**HDL (mg/dl)**	**42.46**	**44.07**	**45.86**	**0.364**
**LDL (mg/dl)**	**86.22**	**95.80**	**60.31**	**0.100**
**Creatinine (mg/dl)**	**1.20**	**1.20**	**0.92**	**0.939**
**AST (U/L)**	**20.48**	**21.25**	**20.53**	**0.384**
**ALT (U/L)**	**24.31**	**19.79**	**20.21**	**0.157**
**ALP (U/L)**	**191.77**	**179.81**	**182.93**	**0.622**
**Hemoglobin (g/dl)**	**13.97**	**13.70**	**14.45**	**0.352**

BMI: Body Mass Index, FBS: Fasting Blood Sugar, SP: Systolic Pressure, DP: Diastolic Pressure, Chol: Cholesterol, TG; Triglyceride, HDL: High Density Lipoprotein, LDL: Low Density Lipoprotein, Statistical significance P < 0.05.

**Table 5:** MTHFR polymorphism and systolic blood pressure (SBP) in diabetic retinopathy patients

**MTHFR C677T**	**CC**	**CT**	**TT**	**P-value**
SP(Mean ± SD)	123.48±12.07	124.93±12.27	130.96±11.92	CC-CT; p>0.05 CC-TT; p=0.01 CT-TT; p>0.05

## Discussion

The present study's findings demonstrated that unlike FVL and *FXIIIVal34Leu* polymorphisms, the difference in the frequency distribution of the *MTHFRC677T* polymorphism was associated with diabetes. The *C677T* variant of *MTHFR* is considered to be the most well-known genetic determinant of folate localization, which resulted in decreased *MTHFR* activity. As a result, homocysteine levels increase that can lead to insulin resistance (11, 12). It has been shown that homocysteine activates vascular inflammation through mediators like vascular endothelial growth factor (13). 

According to previous research, carriers of the *MTHFRC677T* or *677TT* genotypes are more likely to develop dyslipidemia and hypertension, which are important factors associated with metabolic syndrome (14). Similar to our results, carrying the T allele was found to be related to diabetes among Turkish (15), Polish (16), and Indian (17) populations. Settin et al. also stated that the *TT* genotype was associated with T2DM susceptibility (6). A study on the Iranian population showed that *MTHFR* *rs1801131* *A/C* and *rs1801133 T/C* polymorphisms are significantly associated with the risk of T2D (18). These results indicate that the 677TT genotype may be a risk factor in T2DM patients. An independent risk factor for macroangiopathy, hyperhomocysteinemia is caused by a polymorphism mutation (C677T) in the MTHFR gene. Homocysteine stimulates vascular inflammation through inflammatory cytokines, including VEGF (13). Diabetic retinopathy involves a complex association between biochemical and metabolic factors in the retina cells and the precise pathogenesis is not clearly identified (19). 

Although this polymorphism has been suggested that may contribute to the progression of DR, the TT genotype of this polymorphism was significantly lower in our DR population compared to that of T2DM patients. It is due to the fact that various factors affect this phenomenon especially the control of blood glucose by patients. Moreover, homocysteine level was not measured in this study and we do not know the amount of that in our different groups. It is worth mentioning that other factors such as ethnicity and sample size cannot be ignored in making differences in different studies. Regarding the MTHFR genotype, our research showed that the mutant homozygous was also associated with high systolic blood pressure, while the other genotypes were not.

A possible gene for determining the prothrombotic condition found in diabetic retinopathy is coagulation factor XIII, which is implicated in hemostasis, fibrinolysis, vascular remodeling, and tissue healing. The effect of the *FXIIIVal34Leu* polymorphism may change contingent upon the plasma levels of fibrinogen and thrombin in various populations (20). Numerous studies have suggested that fibrinogen is a predictor of atherosclerosis (21, 22). According to these findings, the *Val34* allele on subunit A of factor XIII is associated with increased fibrinogen concentration, leading to increased fibrin clot formation (23). In addition, other studies have shown that this polymorphism is related to both fibrin concentration and insulin resistance (24). *FXIIIVal34Leu* has been associated with thrombotic disorders (25) through altering FXIII specific activity but not FXIII level (26). Indeed, carriers of the *Leu34* allele have been found to have enhanced FXIII activity. As far as we know, no previous study has examined the relationship between the *FXIIIVal34Leu* polymorphism (*rs5985*) and diabetes complications, such as retinopathy. In this study, the case and control groups had slightly higher mutant allele rates than those seen in other Asian populations (27).

Many studies have shown a higher prevalence of *FVL* polymorphism in people with thrombotic diseases (28, 29). Previous studies have examined the prevalence of this polymorphism in various diseases, but so far, the association of *FVL* in patients with diabetic retinopathy has not been evaluated. In a study by Rahimi et al., the prevalence of allele A among those with diabetes who also have coronary artery diseases was reported to be 4.6% (30). In another study performed on patients with gestational diabetes with or without microalbuminuria, the prevalence was 1.6% and 4.9%, respectively, higher than in the present study (31). It should be noted that the frequency of allele A in both studies was not significantly correlated. The prevalence of this allele in type II diabetic patients was not significant in Japanese (32) and Caucasian (33) populations. Due to the existence of such contradictory results, the studies are considered in different societies according to different ethnicities and also with different diseases. The frequency of FVL alleles in the population of western Iran (34) is 2.1% higher than in the present study (1.16%). It is noteworthy to mention that the highest reported allelic frequency so far is related to the Lebanese people (7.88) (35).

As an advantage of the present study was the use of T-ARMS-PCR. This technique is a quick, flexible and affordable SNP detection tool (36). 

Various factors across studies might cause data heterogeneity, including the different characteristics of the populations, discrete data collection methods, different sample sizes, various lifestyles, and different environmental factors and periods of diabetes mellitus (37). Therefore, the results of these studies should be considered with caution. To perform this study, it is recommended to use a larger sample size in different Iranian ethnicities. 

Considering the results of this investigation,* MTHFRC677T* polymorphism is associated with diabetes. Moreover, the TT genotype of this polymorphism is significantly lower in diabetic patients who suffered from retinopathy. Therefore, screening for this polymorphism can be a predictor of diabetes as well as retinopathy in the patients.
